# Risk factors for anaemia among women and their young children hospitalised with suspected thiamine deficiency in northern Lao PDR

**DOI:** 10.1111/mcn.13565

**Published:** 2023-10-06

**Authors:** Sonja Y. Hess, Taryn J. Smith, Dalaphone Sitthideth, Charles D. Arnold, Xiuping Tan, Kerry S. Jones, Kenneth H. Brown, Silvia Alayon, Sengchanh Kounnavong

**Affiliations:** ^1^ Institute for Global Nutrition and Department of Nutrition University of California Davis Davis California USA; ^2^ Lao Tropical and Public Health Institute Vientiane Lao People's Democratic Republic; ^3^ Nutritional Biomarker Laboratory, MRC Epidemiology Unit University of Cambridge Cambridge UK; ^4^ USAID Advancing Nutrition Arlington Virginia USA; ^5^ Save the Children Washington Washington, D.C. USA

**Keywords:** anaemia, iron deficiency, Laos, riboflavin deficiency, thiamine deficiency

## Abstract

Anaemia among women and young children remains a major public health concern. This secondary study describes the anaemia prevalence among young hospitalised children and their mothers in northern Lao People's Democratic Republic and explores possible nutritional causes and risk factors for anaemia. Hospitalised children (ages 21 days to <18 months) with clinical symptoms suggestive of thiamine deficiency disorders were eligible along with their mothers. Venous blood was collected for determination of haemoglobin, ferritin, soluble transferrin receptor (sTfR), retinol‐binding protein (RBP), erythrocyte glutathione reductase activation coefficient (EGRac), thiamine diphosphate (ThDP) and acute phase proteins. Risk factors for anaemia were modelled using minimally adjusted logistic regression controlling for age. Haemoglobin results were available for 436 women (mean ± SD age 24.7 ± 6.4 years; 1.6% pregnant) and 427 children (4.3 ± 3.5 months; 60.3% male). Anaemia prevalence (Hb < 120 g/L for nonpregnant women and <110 g/L for pregnant women and children) was 30.7% among women and 55.2% among children. In bivariate analyses, biomarkers significantly associated with anaemia in women were ferritin, sTfR, RBP, EGRac and ThDP. Other risk factors for women were lower BMI, mid‐upper arm circumference < 23.5 cm, lower education, lower socioeconomic index, food insecurity, Hmong ethnicity, not/rarely having attended antenatal care, not having taken antenatal iron‐containing supplements and not meeting minimum dietary diversity. Risk factors for anaemia among children were older age, male sex, stunting, sTfR, ThDP and alpha‐1‐acid‐glycoprotein. Anaemia was common among women and their hospitalised children and was associated with micronutrient deficiencies and socioeconomic, dietary and health care‐seeking risk factors, suggesting that multiple strategies are required to prevent anaemia among women and children.

## Key messages

- Anaemia is a public health concern among women and young children due to multiple causes and biological, socioeconomic and ecological risk factors.
- Anaemia was assessed among women and their young children hospitalised for clinical signs and symptoms suggestive of thiamine deficiency disorders. The risk of anaemia was significantly associated with multiple indicators of micronutrient status and factors related to poverty and health and dietary practices.
- The present study highlights that anaemia and iron, thiamine, riboflavin and vitamin A deficiencies are highly prevalent in the study population and that multiple strategies are required to prevent anaemia and the other consequences of these micronutrient deficiencies.

## Graphical abstract

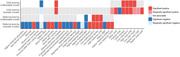

## 1 INTRODUCTION

Anaemia among women of reproductive age and young children is a major public health concern globally (Stevens et al., 2022). Anaemia is the consequence of a wide range of direct causes and underlying risk factors. Iron deficiency is considered the most common nutritional deficiency leading to anaemia, although other micronutrient deficiencies can also contribute (Chaparro & Suchdev, 2019; World Health Organisation [WHO], 2017). In addition, inherited red blood cell disorders, infections such as malaria, hookworm and schistosomiasis, and blood loss also cause anaemia (Hess et al., 2023). Utilisation of health care services, use of prenatal iron folic acid or multiple micronutrient supplements, and socioeconomic factors such as household wealth and educational attainment have also been identified as strong determinants of anaemia at the population level (Owais et al., 2021).

In the Lao People's Democratic Republic (PDR), anaemia remains a major public health concern (Keokenchanh, Kounnavong, Midorikawa, et al., 2021; Keokenchanh, Kounnavong, Tokinobu, et al., 2021). The 2017 national survey found that 39% of women 15–49 years of age and 43% of children 6–59 months of age were anaemic. While risk factors for anaemia differed for women and children, this survey found that higher education of the women or the household head was associated with lower risk of anaemia in both women and children (Keokenchanh, Kounnavong, Midorikawa, et al., 2021; Keokenchanh, Kounnavong, Tokinobu, et al., 2021). Other smaller studies also found a high prevalence of anaemia; for example, 54% of young children 6–23 months of age were anaemic in a study in Khammouane Province (Barffour et al., 2019), and 49% of young children aged 6–52 months were anaemic in Savannakheth Province (Kounnavong et al., 2011). These studies provided important insights into risk factors associated with anaemia, but more studies are needed to support context‐specific targeting of interventions to prevent and manage anaemia.

The recently completed hospital‐based Lao Thiamine Study collected a wealth of information on nutritional, health and socioeconomic status (SES). The study had the primary objective to develop a case definition for thiamine‐responsive disorders among young children with clinical signs and symptoms suggestive of thiamine deficiency disorders (TDD) (Hess et al., 2020). The present study is a secondary analysis aiming to describe the anaemia prevalence and its risk factors among hospitalised infants and young children and their mothers.

## 2 METHODS

### 2.1 Study design, setting and population

The Lao Thiamine Study was implemented in Luang Prabang and surrounding areas of northern Lao PDR. The study protocol was previously published (Hess et al., 2020). Briefly, infants and young children ages 21 days to <18 months who were seeking care at the Lao Friends Hospital for Children for clinical signs and symptoms, such as respiratory and cardiac distress and neurological signs suggestive of TDD, were eligible once written parental consent was obtained. Detailed inclusion and exclusion criteria are described in Supporting Information: Table 1. All primary female caregivers of enroled children were eligible unless acutely ill or unable to provide written consent due to reduced decision‐making ability. The Lao Thiamine Study also included a frequency‐matched community comparison group. However, due to technical problems caused by the delayed haemoglobin assessment in the community, the present study will be limited to the hospital cohort. Since maternal characteristics may determine children's risk for anaemia, we present the results of the women first and then those for children.

### 2.2 Data collection

Self‐reported sociodemographic indicators included information on maternal education, literacy, occupation, household size and composition, housing characteristics, sanitation facilities, source of drinking water and household ownership of assets and land. These proxy indicators were used to estimate a household socioeconomic index using principal component analyses (Vyas & Kumaranayake, 2006). Food security was assessed using the Household Food Insecurity Access Scale (Coates et al., 2007). Mothers of enroled children were interviewed on the number of antenatal care (ANC) contacts, place of delivery and micronutrient supplement use during the pregnancy and lactation of the study child. Minimum dietary diversity for women (MDD‐W) was defined as the consumption of at least 5 of 10 food groups the day before travelling to the hospital (FAO & FHI 360, 2016). Adherence to a restrictive post‐partum taboo diet among women was assessed retrospectively in weekly and monthly intervals since the study child's birth, as previously reported (Smith, Tan, et al., 2022). Infant and young child feeding (IYCF) practices were assessed based on caregiver recall for the day before going to the hospital, in case breastfeeding or dietary intakes were affected by illness or hospitalisation. Reported IYCF practices were used to estimate minimum dietary diversity (MDD), minimum meal frequency (MMF) and minimum acceptable diet (MAD) (WHO, 2021).

Maternal height, weight and left mid‐upper arm circumference (MUAC) and child recumbent length, weight, head circumference and MUAC were assessed following recommended protocols (Cashin & Oot, 2018). Among children, stunting, underweight and wasting were defined as length‐for‐age z‐scores, weight‐for‐age z‐scores and weight‐for‐length z‐scores (WLZ) <‐2 SD, respectively (WHO Multicentre Growth Reference Study Group, 2006). Low MUAC was defined as <23.5 cm for women and <12.5 cm for children (Tang et al., 2013; UNHCR & World Food Programme, 2011).

### 2.3 Blood collection and laboratory analyses

Five millilitres of non‐fasting venous blood was collected in BD vacutainer tubes coated with K2 EDTA from children and 10 mL was collected from women. Blood samples were processed in <1 h. A complete blood count was determined in all study participants using a haematology analyser (BC‐3000 Plus, Shenzhen Mindray Bio‐Medical Electronics Co.). Whole blood, plasma and washed red blood cells were aliquoted as previously described (Hess et al., 2020). Plasma ferritin, soluble transferrin receptor (sTfR), retinol‐binding protein (RBP), C‐reactive protein (CRP) and alpha‐1‐acid‐glycoprotein (AGP) were analysed using the enzyme‐linked immunosorbent assay at the VitMin Lab (Erhardt et al., 2004). Indicators of riboflavin and thiamine status were determined at the Nutritional Biomarker Laboratory, MRC Epidemiology Unit at the University of Cambridge (UK). Specifically, erythrocyte glutathione reductase activation coefficient (EGRac), a functional indicator of riboflavin status, was measured in erythrocytes before and after incubation with added flavin adenine dinucleotide (Public Health England, 2022). The concentration of whole‐blood thiamine diphosphate (ThDP) was determined with high‐performance liquid chromatography with fluorescence detection (Lu & Frank, 2008), and the assay of erythrocyte transketolase activation coefficient (ETKac) was used as a functional indicator of thiamine status (Jones et al., 2021).

Anaemia was defined as haemoglobin <120 g/L for nonpregnant women and <110 g/L for pregnant women and children, respectively (WHO, 2011). For women, mean corpuscular volume (MCV) <80 fL was defined as low and >100 fL was defined as high. For children, age‐specific cut‐offs for low MCV were used; high MCV was not defined as there are no age‐specific cut‐offs available for young children (The Johns Hopkins Hospital et al., 2021). Iron deficiency was defined either based on low ferritin <15 µg/L for women and <12 µg/L for children or high sTfR status (sTfR >8.3 mg/L) (WHO, 2014, 2020). RBP < 0.6 µmol/L was used to define vitamin A deficiency to correspond with a retinol cut‐off of <0.7 µmol/L. There is presently no international consensus for cut‐offs to define riboflavin and thiamine deficiency. Nevertheless, EGRac > 1.30 is widely accepted as indicative of high risk of riboflavin deficiency (Pentieva, 2021) and ETKac > 1.25 as high risk of thiamine deficiency, respectively (Whitfield, 2021). Similarly, there is no consensus for cut‐offs for ThDP, and previous studies used <95 nmol/L among adults (Ihara et al., 2005; Schrijver et al., 1982). Inflammation status was defined as CRP > 5 mg/L and/or AGP > 1 g/L.

### 2.4 Statistical analysis

A statistical analysis plan was developed before initiating analyses (Hess et al., 2022). The altitude of Luang Prabang is 305 m, and WHO does not recommend adjustments for altitude up to 1000 m (WHO, 2011). Although some study participants resided in the mountainous areas surrounding Luang Prabang, haemoglobin concentrations were not adjusted for altitude or smoking. Selected micronutrient status biomarkers were corrected for inflammation following recommendations by the BRINDA Project (Namaste et al., 2019). Specifically, concentrations of ferritin were corrected for women based on all women in the Lao Thiamine Study, and RBP was corrected for children based on the frequency‐matched community cohort because of the high inflammation rate in hospitalised children (Hess et al., 2020). As per BRINDA guidance, other adjustments were not indicated as associations with inflammation markers in this sample were either not present or were in the opposite direction of biologically expected inflammation effects. We excluded thiamine biomarkers for women and children who reportedly received thiamine supplementation before blood draw or had a free thiamine concentration greater than the 90th percentile, a conservative cut‐off.

Logistic regression modelling was used to identify which risk factors were significantly associated with anaemia in minimally adjusted bivariate models, controlling only for child age. Multivariable models were fit including all predictors from bivariate analyses that were associated with anaemia at a level of *p* < 0.1. Because of the reduced sample size for thiamine biomarker results (*n* = 416 women; *n* = 286 children), we repeated the multivariable model with and without thiamine indicators as predictor variables. Multivariable results for thiamine are shown from the reduced sample size model, all other results are presented from the full sample size model. Collinearity was assessed, and if risk factors were correlated, we selected a single variable to represent the relevant information. The purpose of the multivariable model is to assess the relationship between predictor and outcome while controlling for the influence of other factors. If there were significant association based on continuous indicators, we repeated analyses with binary predictors defined by the above‐stated cut‐offs for each indicator and estimated the prevalence of anaemia within those predictor categories to better understand the degree of risk in potentially targetable subgroups. Results are presented as odds ratio (OR) and 95% confidence intervals. All statistical analyses were performed using Stata (version 16.1, StataCorp).

### 2.5 Ethical statement

Ethical approval was obtained from the National Ethics Committee for Health Research, Ministry of Health, Lao PDR, and the Institutional Review Board of the University of California Davis. Written or fingerprint confirmed informed consent from at least one primary caregiver for the child's participation in the study, and from the woman for her own participation, after a detailed explanation of the study in a language appropriate to the family.

## 3 RESULTS

### 3.1 Characteristics of women and their children

**Figure 1:**
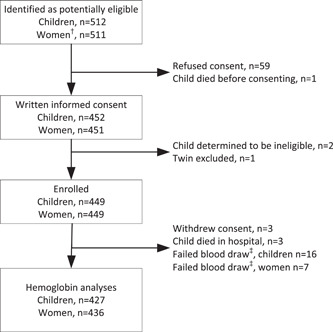
Flow chart of study participants. ^†^Mothers and other female primary caregivers of enroled children were eligible for study participation. When a child dropped out of the study, the same reason for dropout was assigned to both the child and the primary caregiver. ^‡^Failure of blood collection or haemoglobin analyses.

A total of 512 infants and young children were identified as potentially eligible, and 449 women−child dyads were enroled (Figure 1). Haemoglobin results were available for 436 women and 427 children. Women were, on average, 24.7 ± 6.4 years old, 99.5% were biological mothers of the study child, 1.6% were pregnant with another child and average gravidity was 2.7 ± 2 (Table 1). Almost half (44.2%) had low MUAC < 23.5 cm, but only 9.1% had a body mass index (BMI) <18.5 kg/m^2^. Over a third (40.9%) of women reported to have never or very rarely attended ANC during their pregnancy with the study child. Nevertheless, 80.0% reported taking prenatal supplements prescribed by the health centres. As previously reported (Smith, Tan, et al., 2022), the majority of women followed culturally determined dietary restrictions post‐partum either previously (49.3%) or at the time of interview (42.7%), and few women (12.6%) achieved the MDD‐W. Moreover, 39.6% of women reported moderate or severe household food insecurity.

**Table 1:** Characteristics of women and their hospitalised children.

Characteristics	
Women	
*N* a	436
Age (years)	24.7 ± 6.4
Biological mothers, *n* (%)	370 (99.5)
Pregnant, *n* (%)	7 (1.6)
Province of residence, *n* (%)	
Luang Prabang	398 (91.3)
Oudomxay	21 (4.8)
Xayaboury	8 (1.8)
Other	9 (2.1)
Ethnic group, *n* (%)	
Lao	49 (11.7)
Khmu	103 (24.6)
Hmong	267 (63.7)
Education, *n* (%)	
No formal education	103 (24.0)
Some/completed primary	122 (28.4)
Some/completed secondary	185 (43.0)
College/university	20 (4.7)
Occupation, *n* (%)	
Does not work	4 (0.9)
Farmer	306 (71.2)
Unskilled labourer	3 (0.7)
Skilled worker^b^	41 (9.5)
Housewife	75 (17.4)
Other	1 (0.2)
Household SES index	−0.1 ± 1.0
Food insecurity category, *n* (%)	
Mild to none	259 (60.4)
Moderate to severe	170 (39.6)
Gravidity, *n*	2.7 ± 2.0
ANC contacts, *n* (%)	
Never/rarely (0−3 times)	172 (40.9)
Inadequate (4−7 times)	170 (40.4)
Adequate (≥8 times)	79 (18.8)
Reported prenatal supplementation, *n* (%)	344 (80.0)
Location of delivery of infant, *n* (%)	
Hospital	124 (30.8)
Health centre	148 (36.7)
Home	131 (32.5)
Dietary diversity score	3.0 ± 1.4
Achieved MDD‐W,^c^ *n* (%)	54 (12.6)
Follow taboo diet	
Presently restricting diet post‐partum, *n* (%)	186 (42.7)
Previously restricted diet post‐partum, *n* (%)	215 (49.3)
No dietary restrictions post‐partum, *n* (%)	35 (8.0)
Weight (kg)	48.2 ± 6.9
Height (cm)	148.7 ± 5.5
BMI (kg/m^2^)	21.8 ± 2.7
BMI < 18.5 kg/m^2^, *n* (%)	38 (9.1)
MUAC (cm)	24.2 ± 2.6
MUAC < 23.5 cm, *n* (%)	187 (44.2)
Children	
*N*	427
Age (months)	4.3 ± 3.5
Male, *n* (%)	258 (60.3)
Breastfeeding status, *n* (%)	
Exclusive breastfeeding <6 months of age	248 (77.0)
Predominant breastfeeding^d^ <6 months of age	24 (7.5)
Mixed milk feeding^e^ <6 months of age	31 (9.6)
Continued breastfeeding ≥6 months of age	61 (58.7)
No longer breastfeeding, all age groups	34 (8.0)
Complementary feeding indicators, *n* (%)	
Minimum dietary diversity^f^	6 (5.8)
Minimum meal frequency^g^	31 (37.8)
Minimum acceptable diet^g^	1 (1.2)
Length (cm)	59.1 ± 6.8
Weight (kg)	5.5 ± 1.6
MUAC (cm)	12.4 ± 1.5
Head circumference (cm)	39.4 ± 3.1
Length‐for‐age z‐score	−1.6 ± 1.6
Weight‐for‐age z‐score	−1.4 ± 1.5
Weight‐for‐length z‐score	−0.3 ± 1.3
Stunted, *n* (%)	130 (32.8)
Wasted, *n* (%)	45 (11.4)
Underweight, *n* (%)	112 (28.3)

*Note*: Results are shown as mean ± SD for continuous variables and *n* and (percentage) for categorical variables.

Abbreviations: ANC, antenatal care; MDD‐W, minimum dietary diversity for women; MUAC, mid‐upper arm circumference; SES, socioeconomic status.

^a^ Sample size for different outcomes may vary;

^b^ Merchant, business or government employee;

^c^ Consumption of ≥5 out of 10 defined food groups in the previous 24 h/day before going to the hospital was considered as meeting the minimum dietary diversity for women (FAO & FHI 360, 2016);

^d^ Breastfeeding with certain liquids (water, water‐based drinks, fruit juice) among infants <6 months of age; *n* = 322;

^e^ Breastfeeding with formula and/or animal milk (WHO, 2021) among infants <6 months of age; *n* = 322;

^f^ Consumption of ≥5 out of 8 defined food groups in the previous 24 h/day before going to the hospital was considered as meeting the minimum dietary diversity (WHO, 2021); data available for *n* = 104;

^g^ Complementary feeding indicators (WHO, 2021) among 6−18 months study participants; data available for *n* = 82.

The mean age among children was 4.3 ± 3.5 months, with more boys (60.3%) than girls (Table 1). The majority of hospitalised children presented with clinical signs and symptoms consistent with cardiac beriberi, specifically difficulty breathing (66.2%), tachycardia (43.0%) and tachypnoea (36.3%) among the most common (Supporting Information: Table 2). Overall, 69.1% of the children were either exclusively or predominantly breastfed (Table 1). Among children ≥6 months, few (5.8%) achieved MDD.

Mean haemoglobin was 125.3 ± 18.9 g/L among women and 107.3 ± 19.8 g/L among their hospitalised children (Table 2), resulting in an anaemia prevalence of 30.7% among women and 55.2% among children. Anaemia prevalence among children ≥6 months of age was 66.4%. The anaemia prevalence among children was not significantly different by maternal anaemia status among all study participants combined nor among infants <6 months of age (Table 3). However, children ≥6 months of age of anaemic mothers were more likely to be anaemic (*p* = 0.008).

**Table 2:** Haemoglobin, blood indices and indicators of micronutrient status and inflammation among women and their infants and young children in the Lao Thiamine Study.

Characteristics	Women	Children
*N* a	436	427
Blood cell indices		
Haemoglobin (g/L)	125.3 ± 18.9	107.3 ± 19.8
Anaemia, *n* (%)^b^		
Mild anaemia, *n* (%)	61 (14.0)	94 (22.1)
Moderate anaemia, *n* (%)	61 (14.0)	127 (29.8)
Severe anaemia, *n* (%)	12 (2.8)	14 (3.3)
Haematocrit (%)	37.0 ± 4.7	31.3 ± 5.6
Low haematocrit,^c^ *n* (%)	151 (34.6)	202 (47.4)
MCV (fL)	82.1 ± 9.4	80.1 ± 12.1
Low MCV,^d^ *n* (%)	140 (32.1)	202 (47.5)
Elevated MCV (>100 fL), *n* (%)	2 (0.5)	‐
Iron status^e^		
Ferritin, unadjusted (µg/L)	49.7 (15.6−92.0)	165.0 (69.9−220.2)
Ferritin, corrected (µg/L)	29.8 (10.0−53.9)	‐
Low ferritin, corrected,^f^ *n* (%)	136 (31.2)	21 (5.3)
sTfR (mg/L)	5.5 (4.3−8.2)	5.5 (3.8−8.0)
Elevated sTfR (>8.3 mg/L), *n* (%)	108 (24.8)	91 (22.9)
Vitamin A status^e^		
RBP, unadjusted (µmol/L)	1.7 (1.2−2.4)	0.6 (0.4−0.7)
RBP, corrected (µmol/L)	‐	0.6 (0.5−0.8)
Vitamin A deficient (RPB < 0.6 µmol/L), *n* (%)	6 (1.4)	178 (44.1)
Riboflavin status		
EGRac	2.3 (1.9−2.9)	1.5 (1.3−2.0)
High EGRac (>1.3), *n* (%)	428 (97.1)	248 (71.1)
Thiamine status^g^		
Thiamine diphosphate (nmol/L)	70.1 (54.7−88.0)	66.9 (41.4−97.1)
Low thiamine diphosphate (<95 nmol/L), *n* (%)	340 (82.7)	206 (73.6)
ETKac	1.3 (1.2−1.4)	1.2 (1.1−1.5)
High risk of thiamine deficiency (ETKac > 1.25), *n* (%)	246 (59.7)	120 (49.8)
Inflammation status		
CRP (mg/L)	0.8 (0.3−2.0)	5.3 (0.9−22.1)
Elevated CRP (>5 mg/L), *n* (%)	55 (12.6)	202 (50.6)
AGP (g/L)	0.7 (0.6−0.9)	0.9 (0.6−1.4)
Elevated AGP (>1 g/L), *n* (%)	57 (13.1)	160 (40.5)

*Note*: Results are shown as mean ± SD or median (IQR) for continuous variables and n and (percentage) for categorical variables.

Abbreviations: AGP, α‐1‐acid glycoprotein; CRP, C‐reactive protein; EGRac, erythrocyte glutathione reductase activation coefficient; ETKac, erythrocyte transketolase activation coefficient; MCV, mean corpuscular volume; RBP, retinol‐binding protein; sTfR, soluble transferrin receptor

^a^ Sample size for different outcomes may vary;

^b^ Haemoglobin cut‐offs: <120 g/L for nonpregnant women; <110 g/L for pregnant women and children;

^c^ Low haematocrit for adult women: <36%; age‐specific cut‐offs for low haematocrit for children: <1 month, 33%; 1−<2 month, 28%; 2−<6 mo, 31%; 6–24 month, 33% (The Johns Hopkins Hospital et al., 2021);

^d^ Low and elevated MCV for adult women <80 fL and >100 fL; age‐specific cut‐offs for low MCV <1 month, 91 fL; 1−<2 mo, 84 fL; 2−<6 month, 68 fL; 6–24 month, 70 fL (The Johns Hopkins Hospital et al., 2021);

^e^ Corrected for inflammation following recommendations by BRINDA (Namaste et al., 2019). Specifically, concentrations of ferritin corrected for women, and RBP corrected for children;

^f^ Low ferritin (<15 µg/L) for women and (<12 µg/L) for children (WHO, 2020);

^g^ At least one thiamine biomarker result was available for 416 women and 286 children; women and children who received thiamine before blood draw excluded and/or free thiamine concentration >90th percentile.

**Table 3:** Prevalence of anaemia among children by anaemia status of the mother.

Women	Children		Total *n*	*p* Value
	Nonanaemic	Anaemic		
All				0.247
Nonanaemic	138 (47.4)	153 (52.6)	291	
Anaemic	52 (41.3)	74 (58.7)	126	
Total *n*	190	227	417	
Among <6 months of age				0.835
Nonanaemic	107 (49.8)	108 (50.2)	215	
Anaemic	49 (48.5)	52 (51.5)	101	
Total *n*	156	160		
Among ≥6 months of age				0.008
Nonanaemic	31 (40.8)	45 (59.2)	76	
Anaemic	3 (12)	22 (88)	25	
Total *n*	34	67		

*Note*: Results are shown as *n* and (percentage) for categorical variables.

Both biomarkers of iron status suggest a high prevalence of iron deficiency (Table 2). Specifically, 31.2% of women had low ferritin, and 24.8% had elevated sTfR. Moreover, 97.1% of women had EGRac >1.3% and 59.7% had ETKac >1.25, suggesting a high risk of both riboflavin and thiamine deficiencies in the study population. Deficiencies in iron (defined based on sTfR, 22.8%), riboflavin (defined as EGRac >1.3, 71.1%) and thiamine (defined as ETKac >1.25, 49.8%) were also highly prevalent among children (Table 2). Iron deficiency anaemia (low haemoglobin and low ferritin) was 20.6% for women and 4.0% for children. A third of all women (32.6%) and children (32.1%) had three micronutrient deficiencies concurrently. As expected, inflammation was common among the hospitalised children.

### 3.2 Risk factors for anaemia among women and their children based on bivariate analyses

**Figure 2:**
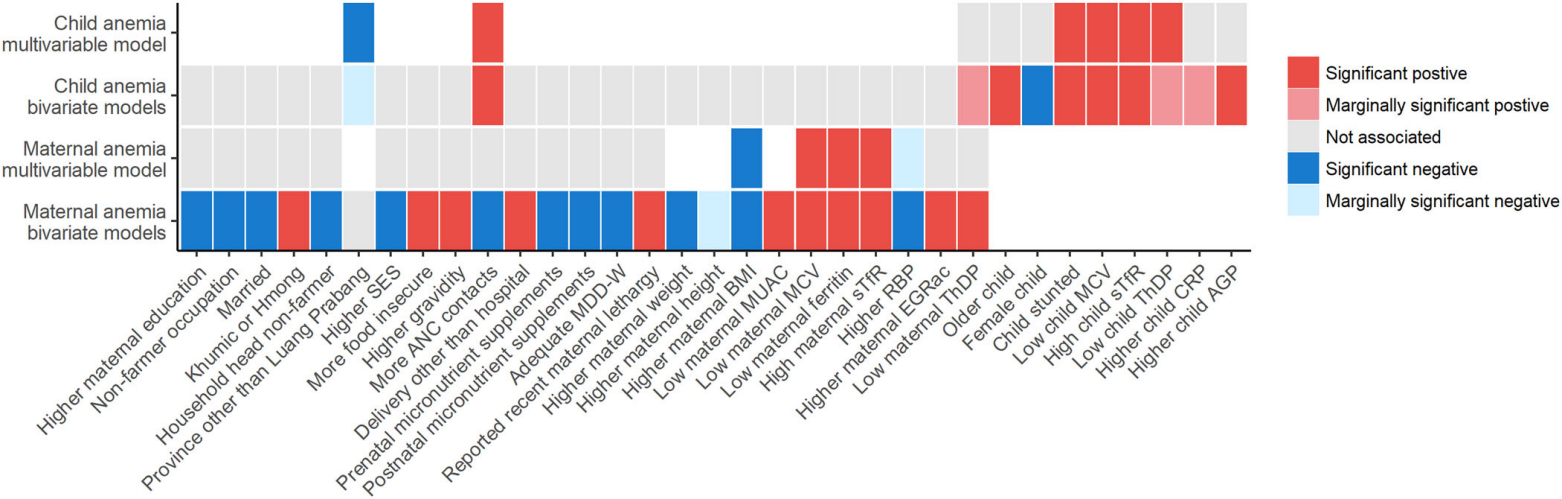
Predictors of anaemia among women and children in bivariate and multivariable logistic regression analyses^†,‡^. ^†^Variables are only shown if they are associated (*p* < 0.1) with the risk of anaemia in at least one of the analyses. If a variable was significantly associated as continuous and dichotomous variable, only the dichotomous result is shown in the figure. Detailed results of all variables tested are available in the Supporting Information: Tables 3−4. ^‡^The variables described as ‘lower’ or ‘higher’ are continuous. The following cut‐offs were used to define dichotomous variables: low maternal MUAC; MUAC < 23.5 cm, low maternal ferritin, ferritin corrected for inflammation <15 µg/L; high maternal or child sTfR, sTfR > 8.3 mg/L; low maternal or child ThDP, <95 nmol/L; stunted, length‐for‐age z‐score < −2 SD; low child MCV, MCV below age‐specific MCV cut‐offs (The Johns Hopkins Hospital et al., 2021). AGP, α‐1‐acid glycoprotein; BMI, body mass index; CRP, C‐reactive protein; EGRac, erythrocyte glutathione reductase activation coefficient; LAZ, length‐for‐age z‐score; MCV, mean corpuscular volume; MDD‐W, minimum dietary diversity of women; MUAC, mid‐upper arm circumference; RBP, retinol‐binding protein; SES, socioeconomic status; sTfR, soluble transferrin receptor; ThDP, thiamine diphosphate.

While women's age was not associated with their risk of anaemia (Figure 2, Supporting Information: Table 3), older children had a higher risk of anaemia (Supporting Information: Table 4). Lower educational attainment among women significantly increased women's risk of anaemia. Compared to Lao ethnicity, maternal Khmuic (OR 3.54, 95% CI 1.28–9.83) and Hmong ethnicity (OR 5.05, 95% CI 1.93–13.2) were associated with 3–5 times increased risk of anaemia among women. Low SES index and household food insecurity both increased the risk of women's anaemia. None of these maternal risk factors were associated with anaemia among their children.

Women who reported having adequate ANC contacts (≥8) during the pregnancy of the study child (*n* = 79) had a lower risk of anaemia (OR 0.22, 95% CI 0.11−0.44) than those who had never or rarely attended ANC (0−3 contacts), and this association was also found for their children (OR 0.78, 95% CI 0.45−1.36). However, children of mothers who reported 4−7 ANC contacts had a higher risk of anaemia (OR 1.62, 95% CI 1.04−2.51) than children of women who attended ANC never or rarely. Reported prenatal supplementation (OR 0.34, 95% CI 0.21−0.55) and current post‐natal micronutrient supplementation (OR 0.39, 95% CI 0.18−0.82) were associated with a reduced anaemia risk among women. Similarly, achieving MDD‐W was associated with less maternal anaemia (OR 0.44, 95% CI 0.21−0.94). In contrast, neither breastfeeding status nor MMF was significantly associated with children's risk of anaemia, and MDD and MAD were too rarely achieved for the model to converge.

Among women, lower BMI (as continuous variable) and low MUAC (<23.5 cm; OR 1.76, 95% CI 1.16−2.68) were both significantly associated with a greater increased risk in maternal anaemia. Among children, stunting was associated with an elevated risk of anaemia (OR 1.90, 95% CI 1.22−2.95).

Among women, indicators of iron status (ferritin, sTfR; *p* < 0.001 for both) and vitamin A status (RBP; *p* < 0.001) were significantly associated with the risk of anaemia (Supporting Information: Table 3). In contrast, only sTfR (*p* = 0.002) was associated with children's anaemia risk (Supporting Information: Table 4). The latter may partly be due to the high level of inflammation among hospitalised children (Table 2). Among children, but not their mothers, AGP concentration was significantly (*p* = 0.043) associated with the risk of anaemia. EGRac was significantly associated only with maternal risk of anaemia (*p* = 0.015). In contrast, ETKac was not associated with anaemia in women or children, but ThDP concentration was significant for both (*p* = 0.001 for women, *p* = 0.025 for children). None of the indicators of maternal micronutrient status were significantly associated with children's risk of anaemia.

### 3.3 Risk factors for anaemia among women and their children based on multivariable logistic regression analyses

Among all the variables associated with anaemia among women in the bivariate analyses with a *p* < 0.1, only BMI (as continuous variable), low MCV, low ferritin and elevated sTfR remained significant in the multivariable logistic regression (Figure 2; Supporting Information: Table 3). Similarly, among children, low MCV and elevated sTfR remained significant in the multivariable logistic regression, along with the province of residence, number of reported ANC contacts, stunting and low ThDP (Supporting Information: Table 4). Estimates and significance of risk factors included in the reduced sample size multivariable model (including ThDP) were similar to that of the full sample (data not shown). While most of these associations were in the directions expected, a higher number of ANC contacts significantly increased the risk of anaemia among children, but not women.

## 4 DISCUSSION

The present cross‐sectional study found a high prevalence of anaemia among women and their infants and young children hospitalised for suggestive TDD. In bivariate analyses among women, many variables were associated with the risk of anaemia; these included biomarkers of iron, vitamin A, riboflavin and thiamine status and a wide range of socioeconomic factors, health‐seeking behaviours, nutrition practices and biological risk factors. Among children, a greater risk of anaemia was associated with older age, male sex, stunting, and indicators of iron and thiamine deficiency and inflammation.

The 2017 national survey in Lao PDR found a weighted anaemia prevalence of 31% among women aged 15−49 years and 31.2% of children 6−59 months of age in northern provinces (Keokenchanh, Kounnavong, Midorikawa, et al. 2021; Keokenchanh, Kounnavong, Tokinobu, et al., 2021). The prevalence among women was comparable to the prevalence in the present study (30.7%), but we found a substantially higher prevalence (66.4%) among hospitalised infants and young children aged 6−18 months. This difference may, in part, be due to the age difference between the children in the two surveys, as older age is generally associated with lower prevalence of anaemia (Engle‐Stone et al., 2017). In addition, it is possible that the anaemia prevalence in the present study was elevated because of the complex interplay of socioeconomic risk factors that may have led both to anaemia and hospitalisation for suggestive TDD (Chaparro & Suchdev, 2019; Smith et al., 2021).

The anaemia prevalence among <6‐month‐old infants was 51.6%. We used the haemoglobin cut‐off <110 g/L recommended for children ≥6 months of age (WHO, 2011) because there are no international cut‐offs for haemoglobin in early infancy. This cut‐off may not be appropriate for younger infants because haemoglobin concentrations tend to be higher in early infancy (The Johns Hopkins Hospital et al., 2021). Thus, among younger infants, it is possible that we underestimated the anaemia prevalence and may have only identified severe cases of anaemia. This is likely the reason why older age was identified as a risk factor for childhood anaemia in the present study, which is in contrast to what is typically found in anaemia surveys (Engle‐Stone et al., 2017).

Iron deficiency was significantly associated with the risk of anaemia among both women and children in the multivariable logistic regression. This is consistent with findings from many studies globally, suggesting that iron deficiency is a primary cause of anaemia (WHO, 2017). Other micronutrient deficiencies contribute to the anaemia burden to a lesser extent (Hess et al., 2023). In the present study, RBP concentration was significantly associated only with women's risk of anaemia, but not children, even though vitamin A deficiency was more prevalent among children. Riboflavin status was significantly associated with anaemia among women, but not children. Although riboflavin is considered a potential cause of anaemia because of its role in erythrocyte production and iron metabolism (WHO, 2017), evidence from cross‐sectional studies and intervention trials has been inconsistent (Aljaadi et al., 2022). In the present study, women's and children's ThDP concentrations were significantly associated with their risk of anaemia in bivariate analyses. Thiamine deficiency is not commonly considered a cause of anaemia (WHO, 2017), except for megaloblastic anaemia responsive to thiamine, which is a rare autosomal recessive hereditary disease with an extremely low incidence (Green & Datta Mitra, 2021). So a more likely explanation for our findings is that anaemia and thiamine deficiency are caused by similar poverty‐related risk factors (Smith, Sitthideth, et al., 2022). This same interaction of poverty‐related risk factors may explain our findings that lower BMI and low MUAC among women and stunting among children were significantly associated with anaemia among women and children, respectively.

The findings of reported gravidity, ANC contacts, prenatal supplementation and location of delivery being all significantly associated with the anaemia risk among women is consistent with previous reports. For example, a recent study found that health care utilisation, especially seeking ANC during pregnancy and the use of contraceptives, were strong drivers for national or subnational decline in anaemia prevalence among women of reproductive age (Owais et al., 2021). In contrast, we found inconsistencies regarding the number of ANC contacts and anaemia risk among children. While the anaemia risk was lower among children whose mother reported an adequate number of ANC contacts (>8), 4−7 ANC contacts were associated with children's greater risk of anaemia. This suggests that the number of ANC contacts was either confounded or not linearly related to childhood anaemia. Overall, our findings highlight the importance of ANC and family planning for reducing the risk of maternal anaemia.

The complex interactions of the various causes and risk factors of anaemia, as well as the cross‐sectional study design, may explain why only few variables remained significant in the multivariable logistic regression. Although we assessed for collinearity, many of the considered health indicators share similar risk factors, which may affect their associations with anaemia in the multivariable models. Analyses of multiple data sources with prospective data collection would be required to identify the most likely causes and underlying risk factors of anaemia.

A weakness of this secondary study is that the study population is not representative of the Luang Prabang region or the country. Indeed, the Lao Thiamine study also included a frequency‐matched community comparison group, and several socioeconomic characteristics and diet and nutrition practices differed significantly from the hospital cohort (Smith, Sitthideth, et al., 2022; Smith, Tan, 2022). While the present study included a wide range of risk factors, only limited information was available on primary causes, such as inherited red blood cell anomalies, infections such as soil‐transmitted helminthiasis, schistosomiasis and malaria, gynaecological and obstetric conditions and other chronic diseases that lead to blood loss, decreased erythropoiesis or destruction of erythrocytes (Chaparro & Suchdev, 2019; Hess et al., 2023; WHO, 2017). Specifically, the lack of information on haemoglobinopathies and other inherited red blood cell anomalies would have been useful considering that these are relatively prevalent in Lao PDR (Tritipsombut et al., 2012). In contrast, malaria is not endemic in northern Lao, and most children were too young for helminth infection. Additionally, the assessment of folate and vitamin B_12_ concentrations would have been interesting due to the sparsity of information in Lao PDR. However, only two women had elevated MCV, suggesting that folate deficiency may be relatively rare in the study area.

In conclusion, the anaemia prevalence was high among women and their hospitalised infants and young children in northern Lao PDR. Several micronutrient deficiencies were associated with the risk of anaemia among women and their children. Moreover, numerous risk factors, many of them poverty‐related, confirm the complex relationships of underlying factors and biological causes of anaemia and suggest that multi‐component and context‐specific interventions are required to prevent anaemia among women and children.

## AUTHOR CONTRIBUTIONS

Sonja Y. Hess, Charles D. Arnold and Kenneth H. Brown conceived and designed the study protocol. Taryn J. Smith and Sonja Y. Hess developed the data collection questionnaires. Sengchanh Kounnavong translated all data collection questionnaires into Lao language. Xiuping Tan programmed all data collection questionnaires. Taryn J. Smith, Dalaphone Sitthideth, Sengchanh Kounnavong and Sonja Y. Hess planned the local study implementation. Taryn J. Smith and Dalaphone Sitthideth supervised the data collection. Kerry S. Jones performed laboratory analysis. Charles D. Arnold performed statistical analyses. Sonja Y. Hess and Charles D. Arnold interpreted the data. Sonja Y. Hess wrote the manuscript. All authors critically reviewed the manuscript and read and approved the final version.

## CONFLICT OF INTEREST STATEMENT

K. H. B., the spouse of S. Y. H., previously worked for the Bill & Melinda Gates Foundation. The remaining authors declare no conflict of interest.

## Supporting information

Supporting information.

## Data Availability

Data underlying the present study is available at https://osf.io/jfke3. Under the grant conditions of the Bill & Melinda Gates Foundation, a Creative Commons Attribution 4.0 Generic License has already been assigned to the Author Accepted Manuscript version that might arise from this submission.

## References

[mcn13565-bib-0001] Aljaadi, A. M. , Devlin, A. M. , & Green, T. J. (2022). Riboflavin intake and status and relationship to anemia. Nutrition Reviews, 81, 114–132. 36018769 10.1093/nutrit/nuac043

[mcn13565-bib-0002] Barffour, M. A. , Hinnouho, G. M. , Kounnavong, S. , Wessells, K. R. , Ratsavong, K. , Bounheuang, B. , Chanhthavong, B. , Sitthideth, D. , Sengnam, K. , Arnold, C. D. , Brown, K. H. , & Hess, S. Y. (2019). Effects of daily zinc, daily multiple micronutrient powder, or therapeutic zinc supplementation for diarrhea prevention on physical growth, anemia, and micronutrient status in rural laotian children: A randomized controlled trial. The Journal of Pediatrics, 207, 80–89.e2. 30580974 10.1016/j.jpeds.2018.11.022 PMC6448681

[mcn13565-bib-0003] Cashin, K. , & Oot, L. (2018). Guide to anthropometry: A practical tool for program planners, managers, and implementers. Food and Nutrition Technical Assistance III Project (FANTA)/FHI 360.

[mcn13565-bib-0004] Chaparro, C. M. , & Suchdev, P. S. (2019). Anemia epidemiology, pathophysiology, and etiology in low‐ and middle‐income countries. Annals of the New York Academy of Sciences, 1450, nyas.14092. 10.1111/nyas.14092 PMC6697587 31008520

[mcn13565-bib-0005] Coates, J. , Swindale, A. , & Bilinsky, P. (2007). Household Food Insecurity Access Scale (HFIAS) for Measurement of Household Food Access: Indicator Guide (v. 3). Food and Nutrition Technical Assistance Project, Academy for Educational Development.

[mcn13565-bib-0006] Engle‐Stone, R. , Aaron, G. J. , Huang, J. , Wirth, J. P. , Namaste, S. M. , Williams, A. M. , Peerson, J. M. , Rohner, F. , Varadhan, R. , Addo, O. Y. , Temple, V. , Rayco‐Solon, P. , Macdonald, B. , & Suchdev, P. S. (2017). Predictors of anemia in preschool children: Biomarkers reflecting inflammation and nutritional determinants of anemia (BRINDA) project. The American Journal of Clinical Nutrition, 106, 402S–415S. 28615260 10.3945/ajcn.116.142323 PMC5490650

[mcn13565-bib-0007] Erhardt, J. G. , Estes, J. E. , Pfeiffer, C. M. , Biesalski, H. K. , & Craft, N. E. (2004). Combined measurement of ferritin, soluble transferrin receptor, retinol binding protein, and C‐reactive protein by an inexpensive, sensitive, and simple sandwich enzyme‐linked immunosorbent assay technique. The Journal of Nutrition, 134, 3127–3132. 15514286 10.1093/jn/134.11.3127

[mcn13565-bib-0008] FAO & FHI 360 . (2016). Minimum dietary diversity for women: A guide for measurement. FAO.

[mcn13565-bib-0009] Green, R. , & Datta Mitra, A. (2021). Anemia resulting from other nutritional deficiencies. In K. Kaushansky , M. A. Lichtman , J. T. Prchal , M. M. Levi , L. J. Burns , & D. Linch , editors, Williams Hematology, 10th Edition. McGraw‐Hill Professional.

[mcn13565-bib-0010] Hess, S. Y. , Owais, A. , Jefferds, M. E. D. , Young, M. F. , Cahill, A. , & Rogers, L. M. (2023). Accelerating action to reduce anemia: Review of causes and risk factors and related data needs. Annals of the New York Academy of Sciences, 1523, 11–23. 36987993 10.1111/nyas.14985 PMC10918744

[mcn13565-bib-0011] Hess, S. Y. , Smith, T. J. , & Arnold, C. D. (2022). *Lao Thiamine Study. Open Science Framework*. https://osf.io/jfke3

[mcn13565-bib-0012] Hess, S. Y. , Smith, T. J. , Fischer, P. R. , Trehan, I. , Hiffler, L. , Arnold, C. D. , Sitthideth, D. , Tancredi, D. J. , Schick, M. A. , Yeh, J. , Stein‐Wexler, R. , McBeth, C. N. , Tan, X. , Nhiacha, K. , & Kounnavong, S. (2020). Establishing a case definition of thiamine responsive disorders among infants and young children in Lao PDR: Protocol for a prospective cohort study. BMJ Open, 10, e036539. 10.1136/bmjopen-2019-036539 PMC7044841 32060165

[mcn13565-bib-0013] Ihara, H. , Hirano, A. , Wang, L. , Okada, M. , & Hashizume, N. (2005). Reference values for whole blood thiamine and thiamine phosphate esters in Japanese adults. Journal of Analytical Bio‐Science, 28, 241–246.

[mcn13565-bib-0014] Jones, K. S. , Parkington, D. A. , Cox, L. J. , & Koulman, A. (2021). Erythrocyte transketolase activity coefficient (ETKAC) assay protocol for the assessment of thiamine status. Annals of the New York Academy of Sciences, 1498, 77–84. 33354793 10.1111/nyas.14547 PMC8451777

[mcn13565-bib-0015] Keokenchanh, S. , Kounnavong, S. , Midorikawa, K. , Ikeda, W. , Morita, A. , & Kitajima, T. (2021). Prevalence of anemia and its associated factors among children aged 6‐59 months in the Lao people's democratic republic: A multilevel analysis. PLoS One, 16, e0248969. 33765048 10.1371/journal.pone.0248969 PMC7993607

[mcn13565-bib-0016] Keokenchanh, S. , Kounnavong, S. , Tokinobu, A. , Midorikawa, K. , Ikeda, W. , & Morita, A. (2021). Prevalence of anemia and its associate factors among women of reproductive age in Lao PDR: Evidence from a nationally representative survey. Anemia, 2021, 8823030. 33520310 10.1155/2021/8823030 PMC7822650

[mcn13565-bib-0017] Kounnavong, S. , Sunahara, T. , Mascie‐Taylor, C. G. N. , Hashizume, M. , Okumura, J. , Moji, K. , Boupha, B. , & Yamamoto, T. (2011). Effect of daily versus weekly home fortification with multiple micronutrient powder on haemoglobin concentration of young children in a rural area, Lao people's democratic republic: A randomised trial. Nutrition Journal, 10, 129. 22111770 10.1186/1475-2891-10-129 PMC3266642

[mcn13565-bib-0018] Lu, J. , & Frank, E. L. (2008). Rapid HPLC measurement of thiamine and its phosphate esters in whole blood. Clinical Chemistry, 54, 901–906. 18356241 10.1373/clinchem.2007.099077

[mcn13565-bib-0019] Namaste, S. M. L. , Ou, J. , Williams, A. M. , Young, M. F. , Yu, E. X. , & Suchdev, P. S. (2019). Adjusting iron and vitamin A status in settings of inflammation: A sensitivity analysis of the biomarkers reflecting inflammation and nutritional determinants of anemia (BRINDA) approach. American Journal of Clinical Nutrition, 112, 458s–467s. 32743650 10.1093/ajcn/nqaa141 PMC7396268

[mcn13565-bib-0020] Owais, A. , Merritt, C. , Lee, C. , & Bhutta, Z. A. (2021). Anemia among women of reproductive age: An overview of global burden, trends, determinants, and drivers of progress in low‐ and Middle‐Income countries. Nutrients, 13, 2745. 34444903 10.3390/nu13082745 PMC8401240

[mcn13565-bib-0021] Pentieva, K. (2021). Riboflavin. In R. S. Gibson (Ed.), Principles of Nutritional Assessment (3rd edition).

[mcn13565-bib-0022] Public Health England (2022). *National Diet and Nutrition Survey Rolling Programme Years 9‐11 (2016/2017 to 2018/2019). Appendix Q: Methods of blood and urinary analysis and quality control (QC) and quality assessment (QA)*. https://www.gov.uk/government/statistics/ndns-results-from-years-9-to-11-2016-to-2017-and-2018-to-2019

[mcn13565-bib-0023] Schrijver, J. , Speek, A. J. , Klosse, J. A. , Van Rijn, H. J. M. , & Schreurs, W. H. P. (1982). A reliable semiautomated method for the determination of total thiamine in whole blood by the thiochrome method with high‐performance liquid chromatography. Annals of Clinical Biochemistry: International Journal of Laboratory Medicine, 19, 52–56. 10.1177/000456328201900111 7065633

[mcn13565-bib-0024] Smith, T. J. , Johnson, C. R. , Koshy, R. , Hess, S. Y. , Qureshi, U. A. , Mynak, M. L. , & Fischer, P. R. (2021). Thiamine deficiency disorders: A clinical perspective. Annals of the New York Academy of Sciences, 1498, 9–28. 33305487 10.1111/nyas.14536 PMC8451766

[mcn13565-bib-0025] Smith, T. J. , Sitthideth, D. , Tan, X. , Arnold, C. D. , Kounnavong, S. , & Hess, S. Y. (2022). Nutrition and health‐seeking practices during pregnancy and lactation and potential strategies to increase micronutrient intakes among women in Northern Lao PDR. Journal of Nutritional Science, 11, e95. 36405099 10.1017/jns.2022.94 PMC9641509

[mcn13565-bib-0026] Smith, T. J. , Tan, X. , Arnold, C. D. , Sitthideth, D. , Kounnavong, S. , & Hess, S. Y. (2022). Traditional prenatal and postpartum food restrictions among women in Northern Lao PDR. Maternal & child nutrition, 18, e13273. 34595830 10.1111/mcn.13273 PMC8710103

[mcn13565-bib-0027] Stevens, G. A. , Paciorek, C. J. , Flores‐Urrutia, M. C. , Borghi, E. , Namaste, S. , Wirth, J. P. , Suchdev, P. S. , Ezzati, M. , Rohner, F. , Flaxman, S. R. , & Rogers, L. M. (2022). National, regional, and global estimates of anaemia by severity in women and children for 2000–19: A pooled analysis of population‐representative data. The Lancet Global Health, 10, e627–e639. 35427520 10.1016/S2214-109X(22)00084-5 PMC9023869

[mcn13565-bib-0028] Tang, A. M. , Dong, K. , Deitchler, M. , Chung, M. , Maalouf‐Manasseh, Z. , & Tumilowicz, A. (2013). Use of cutoffs for mid‐upper arm circumference (MUAC) as an indicator or predictor of nutritional and health‐related outcomes in adolescents and adults: A systematic review. FANTA, FHI 360.

[mcn13565-bib-0029] The Johns Hopkins Hospital , Kleinman, K. , McDaniel, L. , & Molloy, M. (2021). The Harriet Lane Handbook (22nd edition.). Philadelphia, PA: Elsevier.

[mcn13565-bib-0030] Tritipsombut, J. , Sanchaisuriya, K. , Phollarp, P. , Bouakhasith, D. , Sanchaisuriya, P. , Fucharoen, G. , Fucharoen, S. , & Schelp, F. P. (2012). Micromapping of thalassemia and hemoglobinopathies in diferent regions of northeast Thailand and vientaine, laos people's democratic republic. Hemoglobin, 36, 47–56. 22122810 10.3109/03630269.2011.637149

[mcn13565-bib-0031] UNHCR & World Food Programme . (2011). Guidelines for selective feeding: the management of malnutrition in emergencies. United Nations High Commissioner for Refugees.

[mcn13565-bib-0032] Vyas, S. , & Kumaranayake, L. (2006). Constructing socio‐economic status indices: How to use principal components analysis. Health Policy and Planning, 21, 459–468. 17030551 10.1093/heapol/czl029

[mcn13565-bib-0033] Whitfield, K. C. (2021). Thiamine. In R. S. Gibson (Ed.), Principles of Nutritional Assessment (3rd edition).

[mcn13565-bib-0034] WHO Multicentre Growth Reference Study Group . (2006). WHO Child Growth Standards: Length/height‐for‐age, weight‐for‐age, weight‐for‐length, weight‐for‐height and body mass index‐for‐age: Methods and development. World Health Organization.

[mcn13565-bib-0035] World Health Organization . (2011). Haemoglobin concentrations for the diagnosis of anaemia and assessment of severity, In *Vitamin and Mineral Nutrition Information System*, pp. WHO/NMH/NHD/MNM/11.11. World Health Organization.

[mcn13565-bib-0036] World Health Organization . (2014). Serum transferrin receptor levels for the assessment of iron status and iron deficiency in populations, In *Vitamin and Mineral Nutrition Information System*, pp. WHO/NMH/NHD/EPG/14.16. World Health Organization.

[mcn13565-bib-0037] World Health Organization . (2017). Nutritional anaemias: Tools for effective prevention and control. World Health Organization.

[mcn13565-bib-0038] World Health Organization . (2020). WHO guideline on use of ferritin concentrations to assess iron status in individuals and populations. World Health Organization. 33909381

[mcn13565-bib-0039] World Health Organization . (2021). Indicators for assessing infant and young child feeding practices: definitions and measurement methods. World Health Organization and the United Nations Children's Fund (UNICEF).

