# Supplementary material for: Risk factors for anaemia among women and their young children hospitalised with suspected thiamine deficiency in northern Lao PDR
Source: Matern Child Nutr. 2023 Oct 6;20(1):e13565. doi: 10.1111/mcn.13565 (PMC10749997; doi:10.1111/mcn.13565)
Supplement: Supplementary file 1 — Supporting information. [file MCN-20-e13565-s001.pdf]

Supplementary Material

Risk factors for anemia among women and their young children hospitalized with suspected thiamine deficiency in northern Lao PDR

Sonja Y Hess, Taryn J Smith, Dalaphone Sitthideth, Charles D Arnold, Xiuping Tan, Kerry S Jones, Kenneth H Brown, Silvia Alayon, Sengchanh Kounnavong

Supplementary Table 1: Inclusion and exclusion criteria for infants and young children and their mothers participating in the Lao Thiamine Study

Study group	Inclusion and exclusion criteria
Hospitalized children	*Inclusion criteria:*<br>- Written parental consent<br>- Target age range: 21 days to <18 months<br><br>Plus at least one of the following inclusion criteria:<br>- Liver enlargement (>2 cm below right costal margin on calm, supine exam)<br>- Edema<br>- Tachypnea (> 60/min for 3-8 wks; > 50/min for 2-11 mo; > 40/min for 12-18 mo)<br>- Tachycardia (heart rate >160/min for <12 mo; >120/min for 12 mo - 18 mo)<br>- Oxygen saturation < 92%<br>- Difficulty breathing (i.e. chest in-drawing, nasal flaring)<br>- Refusal to breastfeed or refusal of infant formula or food for greater than 24 hours<br>- Repetitive or recurring vomiting with no obvious other cause (i.e. vomiting >3 times in past 24 hours)<br>- Persistent crying not relieved by soothing and feeding with no obvious other cause<br>- Hoarse voice / cry or loss of voice<br>- Nystagmus or other unusual eye movement<br>- Muscle twitching<br>- Loss of consciousness<br>- Convulsion<br>- Opisthotonus / abnormal posturing<br>- Acute paralysis / flaccid paralysis
Mothers[1] of hospitalized children	*Inclusion criteria:*<br>- Written informed consent<br>*Exclusion criteria:*<br>- Severe acute illness warranting immediate hospital referral<br>- Unable to provide informed consent due to reduced decision-making ability
Community-based children	*Inclusion criteria:*<br>- Written parental consent<br>- Frequency matched to hospitalized children based on age, sex and location of residence<br>*Exclusion criteria:*<br>- Severe acute illness warranting immediate hospital referral
Mothers[1] of community-based children	*Inclusion criteria:*<br>- Written informed consent<br>*Exclusion criteria:*<br>- Severe acute illness warranting immediate hospital referral<br>- Unable to provide informed consent due to reduced decision-making ability

[1] All mothers or other female primary caregivers of enrolled children in the hospital and community were eligible

Supplementary Table 2: Clinical signs and symptoms at enrollment[1] among 427 infants and young children in the hospital cohort of the Lao Thiamine Study

	N	%
Difficulty breathing	297	66.2
Tachycardia	193	43.0
Persistent crying not relieved by soothing or feeding with no obvious other cause	171	38.1
Tachypnea / rapid breathing	163	36.3
Oxygen saturation less than 92%	159	35.4
Hoarse voice / cry or loss of voice	155	34.5
Liver enlarged more than 2 cm[2]	125	27.8
Refusal to breastfeed or refusal of infant formula or food for more than 24 hours	94	20.9
Repetitive or recurring vomiting in past 24 hours with no obvious other cause	89	19.8
Convulsion	43	9.6
Loss of consciousness	13	2.9
Opisthotonus /Abnormal posturing[3]	10	2.2
Muscle twitching	10	2.2
Edema	9	2.0
Nystagmus or other unusual eye movement	9	2.0
Paralysis[3]	1	0.2

[1] Clinical signs and symptoms suggestive of thiamine deficiency disorders, which served as inclusion criteria for infants and young children 3 weeks - <18 months of age

[2] n=120 (27%) unknown

[3] n=121 (27%) unknown

Supplemental Table 3: Risk factors for maternal anemia

	N	Anemia prevalence (%)	Bivariate Odds ratio (95% CI)	Bivariate p-value	Multivariable Odds ratio (95% CI)	Multivariable p-value
Maternal age (yr)	436		1.01 (0.97, 1.04)	0.722		
Mother completed primary education				0.000		0.459
Incomplete primary	169	42.6	ref.		ref.	
Completed primary	261	23	0.4 (0.26, 0.61)		0.72 (0.31, 1.70)	
Maternal occupation				0.005		0.279
Farmer (cultivation and livestock)	309	35.6	ref.		ref.	
Skilled laborer	41	2.4	0.05 (0.01, 0.35)		0.15 (0.01, 2.28)	
Housewife	79	26.6	0.66 (0.38, 1.15)		1.32 (0.43, 4.02)	
Maternal marital status				0.026		0.470
Cohabitation	113	38.9	ref.		ref.	
Married	311	27.7	0.6 (0.38, 0.94)		0.73 (0.31, 1.72)	
Maternal ethnic group				0.003		0.259
Lao	49	10.2	ref.		ref.	
Khmuic	103	29.1	3.54 (1.28, 9.83)		2.32 (0.57, 9.42)	
Hmong	267	35.6	5.05 (1.93, 13.2)		1.16 (0.28, 4.75)	
Household head occupation				0.001		0.525
Farmer (cultivation and livestock)	313	36.1	ref.		ref.	
Skilled laborer	79	12.7	0.26 (0.13, 0.53)		0.56 (0.17, 1.89)	
Housewife	33	27.3	0.7 (0.31, 1.57)		1.16 (0.30, 5.29)	
District of residence				0.129		
Xay	8	37.5	ref.			
Nga	7	85.7	8.56 (0.66, 111.85)			
Luang Prabang	74	16.2	0.29 (0.06, 1.39)			
Xieng ngeun	46	37	0.85 (0.18, 4.09)			
Nan	12	25	0.49 (0.07, 3.45)			
Park ou	68	33.8	0.78 (0.17, 3.61)			
Nambak	60	35	0.79 (0.17, 3.71)			
Ngoi	16	18.8	0.32 (0.05, 2.2)			
Pak xeng	17	29.4	0.64 (0.11, 3.8)			
Phonxay	28	28.6	0.58 (0.11, 3.08)			
Chomphet	41	41.5	1.01 (0.21, 4.92)			

	N	Anemia prevalence (%)	Bivariate Odds ratio (95% CI)	Bivariate p-value	Multivariable Odds ratio (95% CI)	Multivariable p-value
Viengkham	15	20	0.34 (0.05, 2.35)			
Phoukhoune	17	29.4	0.62 (0.1, 3.68)			
Other, specify	27	29.6	0.67 (0.13, 3.53)			
Province				0.148		
Luangprabang	398	29.9	ref.			
Other province	38	39.5	1.67 (0.83, 3.35)			
Estimated time travel from Luang Prabang	430		1.02 (0.91, 1.15)	0.764		
Number of household members	430		0.98 (0.92, 1.04)	0.490		
Number of children under 5y	430		1.03 (0.86, 1.24)	0.733		
SES index	428		0.56 (0.43, 0.72)	0.000	1.23 (0.73, 2.05)	0.439
Food insecurity category				0.021		0.309
None	159	22	ref.		ref.	
Mild	100	32	1.65 (0.94, 2.9)		0.48 (0.18, 1.25)	
Moderate	72	36.1	1.99 (1.08, 3.67)		1.22 (0.42, 3.53)	
Severe	98	39.8	2.29 (1.32, 3.98)		0.75 (0.26, 2.16)	
Number of times mother has been pregnant	430		1.15 (1.04, 1.27)	0.007	1.15 (0.97, 1.38)	0.114
ANC visits				0.000		0.424
Never/very rarely (0-3)	172	44.8	ref.		ref.	
Inadequate (4-7)	170	24.7	0.4 (0.25, 0.63)		0.88 (0.36, 2.11)	
Adequate (>=8)	79	15.2	0.22 (0.11, 0.44)		0.48 (0.15, 1.55)	
Location of delivery of infant				0.000		0.899
Hospital	124	19.4	ref.		ref.	
Health center	148	28.4	1.63 (0.92, 2.89)		1.12 (0.46, 2.72)	
Home	131	45.8	3.44 (1.96, 6.05)		0.92 (0.34, 2.50)	
Mother takes vitamin supplement during pregnancy				0.000		0.997
No	85	50.6	ref.		ref.	
Yes	344	25.9	0.34 (0.21, 0.55)		1.00 (0.38, 2.64)	
Mother currently takes vitamin supplement while breastfeeding				0.014		0.401
No	374	32.9	ref.		ref.	
Yes	55	16.4	0.39 (0.18, 0.82)		0.61 (0.20, 1.91)	
Minimum dietary diversity - women				0.033		0.181

	N	Anemia prevalence (%)	Bivariate Odds ratio (95% CI)	Bivariate p-value	Multivariable Odds ratio (95% CI)	Multivariable p-value
No	376	32.7	ref.		ref.	
Yes	54	16.7	0.44 (0.21, 0.94)		0.43 (0.12, 1.48)	
During pregnancy did you at any point follow a taboo diet?				0.219		
No	418	30.4	ref.			
Yes	10	50	2.2 (0.62, 7.76)			
Did you follow a taboo diet after the birth of your baby?				0.444		
No	6	16.7	ref.			
Yes	422	31	2.32 (0.27, 20.13)			
To date, have you resumed eating your normal diet?				0.460		
No	115	35.7	ref.			
Yes	307	29.3	0.83 (0.51, 1.35)			
Do you currently breastfeed your child?				0.632		
No	30	23.3	ref.			
Yes	406	31.3	1.25 (0.5, 3.09)			
Mother has tingling or loss of sensation in hands or feet in the past 2 weeks				0.484		
No	279	29.4	ref.			
Yes	151	33.1	1.16 (0.76, 1.79)			
Mother felt lethargic in the past 2 weeks				0.031		0.787
No	291	27.1	ref.		ref.	
Yes	139	38.1	1.61 (1.05, 2.48)		1.11 (0.52, 2.37)	
Mother felt nauseous in the past 2 weeks				0.204		
No	372	29.6	ref.			
Yes	58	37.9	1.45 (0.82, 2.59)			
Mother has vomiting in the past 2 weeks				0.963		
No	403	30.8	ref.			
Yes	27	29.6	0.98 (0.42, 2.31)			
Mother has a reduced appetite in the past 2 weeks				0.129		
No	333	28.8	ref.			
Yes	97	37.1	1.45 (0.9, 2.33)			
Mother has low mood or depression in the past 2 weeks				0.828		
No	370	30.8	ref.			
Yes	60	30	0.94 (0.52, 1.7)			

	N	Anemia prevalence (%)	Bivariate Odds ratio (95% CI)	Bivariate p-value	Multivariable Odds ratio (95% CI)	Multivariable p-value
Mother has sleep problems in the past 2 weeks				0.646		
No	287	31.4	ref.			
Yes	143	29.4	0.9 (0.58, 1.4)			
Mother has any short- or long-term memory problems in the past 2 weeks				0.738		
No	328	31.1	ref.			
Yes	102	29.4	0.92 (0.57, 1.5)			
Maternal weight (kg)	423		0.94 (0.91, 0.97)	0.000		
Maternal height (cm)	419		0.97 (0.93, 1)	0.079		
Maternal BMI (kg/m2)	419		0.87 (0.8, 0.95)	0.002	0.86 (0.75, 0.97)	0.019
BMI<18.5				0.289		
No	381	29.9	ref.			
Yes	38	36.8	1.46 (0.72, 2.95)			
Maternal MUAC (cm)	423		0.86 (0.79, 0.94)	0.001		
MUAC <23.5 cm				0.008		
No	236	25	ref.			
Yes	187	37.4	1.76 (1.16, 2.68)			
Mean Corpuscular Volume (MCV)	436		0.86 (0.84, 0.89)	0.000		
MCV <80 fL				0.000		0.000
No	296	15.9	ref.		ref.	
Yes	140	62.1	8.88 (5.57, 14.15)		5.01 (2.44,10.29)	
MCV >100 fL				0.588		
No	434	30.6	ref.			
Yes	2	50	2.16 (0.13, 34.94)			
FERritin, corrected (Women)	436		0.96 (0.95, 0.97)	0.000		
Low corrected ferritin(<15) (Women)				0.000		0.000
No	300	14.7	ref.		ref.	
Yes	136	66.2	12.67 (7.71, 20.81)		4.99 (2.13, 11.67)	
sTfR mg/L	436		1.55 (1.41, 1.7)	0.000		

	N	Anemia prevalence (%)	Bivariate Odds ratio (95% CI)	Bivariate p-value	Multivariable Odds ratio (95% CI)	Multivariable p-value
Elevated sTFR(>8.3) (Women)				0.000		0.015
No	328	17.4	ref.		ref.	
Yes	108	71.3	11.78 (7.1, 19.56)		3.16 (1.26, 7.97)	
RBP umol/L	436		0.47 (0.35, 0.62)	0.000	0.65 (0.42, 1.02)	0.062
Vitamin A deficient- RBP<0.6 (Women)				0.334		
No	430	30.5	ref.			
Yes	6	50	2.22 (0.44, 11.14)			
EGRAC ratio	436		1.4 (1.07, 1.84)	0.015	1.36 (0.9, 2.06)	0.149
High EGRac (>1.3) (Women)				0.553		
No	13	23.1	ref.			
Yes	423	31	1.49 (0.40, 5.52)			
ETKAC	412		1.93 (0.7, 5.38)	0.206		
High ETKac (>1.25) (Women)				0.290		
No	166	28.9	ref.			
Yes	246	33.3	1.26 (0.82, 1.94)			
Blood ThdP nmol/L	411		0.99 (0.98, 0.99)	0.001		
Low thiamine diphosphate (<95 nmol/L)				0.018		0.169
No	71	19.7	ref.		ref.	
Yes	340	33.8	2.13 (1.14, 4)		1.86 (0.7, 4.95)	
CRP mg/L	436		1.01 (0.98, 1.04)	0.566		
CRP >5 mg/L (Women)				0.961		
No	381	30.7	ref.			
Yes	55	30.9	0.98 (0.53, 1.82)			
AGP g/L	436		1.37 (0.65, 2.85)	0.406		
AGP > 1 g/L (Women)				0.219		
No	379	29.6	ref.			
Yes	57	38.6	1.44 (0.81, 2.57)			

	N	Anemia prevalence (%)	Bivariate Odds ratio (95% CI)	Bivariate p-value	Multivariable Odds ratio (95% CI)	Multivariable p-value

Because of the reduced sample size for thiamine biomarker results, we repeated the multivariable model with and without thiamine outcomes. Multivariable results for thiamine are shown in this reduced sample model, all other results are presented in the full sample size model.

Supplemental Table 4: Risk factors for child anemia

	N	Anemia prevalence (%)	Bivariate Odds ratio (95% CI)	Bivariate p-value	Multivariable Odds ratio (95% CI)	Multivariable p-value
Maternal age (yr)	426		1.00 (0.97, 1.03)	0.850		
Mother completed primary education				0.315		
Incomplete primary	166	51.8	ref.			
Completed primary	252	56.7	1.23 (0.82, 1.82)			
Maternal occupation				0.769		
Farmer (cultivation and livestock)	303	55.4	ref.			
Skilled laborer	40	52.5	0.79 (0.4, 1.55)			
Housewife	74	54.1	0.92 (0.55, 1.53)			
Maternal marital status				0.385		
Cohabitation	105	51.4	ref.			
Married	305	55.7	1.22 (0.78, 1.91)			
Maternal ethnic group				0.342		
Lao	49	63.3	ref.			
Khmuic	98	56.1	0.75 (0.37, 1.53)			
Hmong	261	53.3	0.64 (0.34, 1.2)			
Household head occupation				0.124		
Farmer (cultivation and livestock)	307	57.3	ref.			
Skilled laborer	76	46.1	0.6 (0.36, 1.01)			
Housewife	33	51.5	0.71 (0.34, 1.47)			
District of residence				0.280		
Xay	8	62.5	ref.			
Nga	7	28.6	0.3 (0.03, 2.72)			
Luang Prabang	74	52.7	0.78 (0.17, 3.63)			
Xieng ngeun	46	45.7	0.58 (0.12, 2.82)			
Nan	12	75	2.18 (0.3, 15.6)			
Park ou	68	54.4	0.81 (0.17, 3.78)			
Nambak	57	54.4	0.85 (0.18, 4.04)			
Ngoi	16	56.3	1 (0.17, 5.89)			
Pak xeng	15	80	2.7 (0.39, 18.83)			
Phonxay	28	71.4	1.83 (0.34, 9.86)			
Chomphet	39	61.5	1.2 (0.24, 5.96)			
Viengkham	13	53.8	0.95 (0.15, 5.95)			
Phoukhoune	15	40	0.47 (0.08, 2.81)			

	N	Anemia prevalence (%)	Bivariate Odds ratio (95% CI)	Bivariate p-value	Multivariable Odds ratio (95% CI)	Multivariable p-value
Other, specify	28	46.4	0.55 (0.11, 2.86)			
Province				0.095		0.024
Luangprabang	388	56.2	ref.		ref.	
Other province	38	44.7	0.56 (0.28, 1.11)		0.38 (0.16, 0.88)	
Estimated time travel from Luang Prabang	420		0.93 (0.83, 1.05)	0.237		
Number of household members	419		1.04 (0.98, 1.1)	0.236		
Number of children under 5y	419		1.16 (0.97, 1.39)	0.102		
SES index	417		0.93 (0.76, 1.14)	0.486		
Food insecurity category				0.669		
None	152	52	ref.			
Mild	102	54.9	1.16 (0.7, 1.93)			
Moderate	71	57.7	1.29 (0.73, 2.3)			
Severe	93	58.1	1.36 (0.8, 2.3)			
Number of times mother has been pregnant	415		1 (0.9, 1.1)	0.972		
ANC visits				0.020		0.029
Never/very rarely (0-3)	165	51.5	ref.		ref.	
Inadequate (4-7)	168	62.5	1.62 (1.04, 2.51)		1.71 (1.03, 2.84)	
Adequate (>=8)	74	45.9	0.78 (0.45, 1.36)		0.77 (0.4, 1.51)	
Location of delivery of infant				0.609		
Hospital	122	59	ref.			
Health center	143	52.4	0.78 (0.48, 1.28)			
Home	127	52.8	0.83 (0.5, 1.38)			
Mother takes vitamin supplement during pregnancy				0.850		
No	87	54	ref.			
Yes	331	55.3	1.05 (0.65, 1.69)			
Mother currently takes vitamin supplement while breastfeeding				0.402		
No	368	54.3	ref.			
Yes	50	60	1.3 (0.71, 2.38)			
Minimum dietary diversity - women				0.960		
No	365	54.5	ref.			
Yes	54	57.4	0.98 (0.54, 1.78)			
During pregnancy did you at any point follow a taboo diet?				0.251		
No	404	55.2	ref.			
Yes	9	33.3	0.44 (0.11, 1.79)			

	N	Anemia prevalence (%)	Bivariate Odds ratio (95% CI)	Bivariate p-value	Multivariable Odds ratio (95% CI)	Multivariable p-value
Did you follow a taboo diet after the birth of your baby?				0.327		
No	7	71.4	ref.			
Yes	406	54.4	0.44 (0.08, 2.29)			
To date, have you resumed eating your normal diet?				0.170		
No	110	44.5	ref.			
Yes	296	58.1	1.39 (0.87, 2.24)			
Do you currently breastfeed your child?				0.505		
No	32	56.3	ref.			
Yes	390	54.9	1.3 (0.6, 2.81)			
Mother has tingling or loss of sensation in hands or feet in the past 2 weeks				0.830		
No	267	54.7	ref.			
Yes	148	54.7	1.05 (0.7, 1.57)			
Mother felt lethargic in the past 2 weeks				0.813		
No	279	54.8	ref.			
Yes	136	54.4	1.05 (0.69, 1.6)			
Mother felt nauseous in the past 2 weeks				0.379		
No	358	55.6	ref.			
Yes	57	49.1	0.78 (0.44, 1.37)			
Mother has vomiting in the past 2 weeks				0.244		
No	388	53.9	ref.			
Yes	27	66.7	1.64 (0.71, 3.77)			
Mother has a reduced appetite in the past 2 weeks				0.333		
No	321	56.1	ref.			
Yes	94	50	0.8 (0.5, 1.27)			
Mother has low mood or depression in the past 2 weeks				0.975		
No	357	54.9	ref.			
Yes	58	53.4	0.99 (0.57, 1.74)			
Mother has sleep problems in the past 2 weeks				0.419		
No	275	53.5	ref.			
Yes	140	57.1	1.19 (0.78, 1.8)			
Mother has any short- or long-term memory problems in the past 2 weeks				0.294		
No	317	53.3	ref.			
Yes	98	59.2	1.28 (0.81, 2.04)			
Maternal weight (kg)	407		0.99 (0.96, 1.02)	0.408		

	N	Anemia prevalence (%)	Bivariate Odds ratio (95% CI)	Bivariate p-value	Multivariable Odds ratio (95% CI)	Multivariable p-value
Maternal height (cm)	405		0.99 (0.96, 1.03)	0.622		
Maternal BMI (kg/m2)	405		0.98 (0.91, 1.05)	0.552		
Maternal BMI<18.5				0.234		
No	367	53.4	ref.			
Yes	38	65.8	1.54 (0.76, 3.14)			
Maternal MUAC (cm)	407		0.97 (0.9, 1.05)	0.496		
Maternal MUAC <23.5 cm				0.547		
No	228	56.6	ref.			
Yes	179	52.5	0.88 (0.59, 1.32)			
Maternal Mean Corpuscular Volume (MCV)	418		0.99 (0.97, 1.01)	0.400		
Maternal MCV <80 fL				0.984		
No	282	54.6	ref.			
Yes	136	54.4	1 (0.66, 1.51)			
FERritin, corrected (Women)	419		1 (1, 1.01)	0.193		
Low corrected ferritin(<15) - women				0.524		
No	287	55.4	ref.			
Yes	132	53	0.87 (0.57, 1.33)			
Maternal sTfR mg/L	419		1 (0.94, 1.05)	0.864		
Elevated sTFR(>8.3) - women				0.535		
No	315	54	ref.			
Yes	104	56.7	1.15 (0.73, 1.81)			
Maternal RBP umol/L	419		0.94 (0.75, 1.19)	0.609		
Vitamin A deficient- RBP<0.6 (Women)				0.514		
No	413	54.5	ref.			
Yes	6	66.7	1.77 (0.32, 978)			
Maternal EGRAC ratio	419		1.05 (0.81, 1.37)	0.696		
Maternal High EGRac (>1.3) (Women)				0.9343		
No	13	53.8	ref.			
Yes	406	54.7	1.05 (0.34, 3.23)			
Maternal ETKAC	397		1.93 (0.7, 5.38)	0.206		
High ETKac (>1.25) (Women)				0.613		
No	163	52.1	ref.			
Yes	234	56	1.11 (0.74, 1.67)			
Maternal Blood ThdP nmol/L	396		1 (0.99, 1)	0.471		

	N	Anemia prevalence (%)	Bivariate Odds ratio (95% CI)	Bivariate p-value	Multivariable Odds ratio (95% CI)	Multivariable p-value
Maternal Low thiamine diphosphate (<95 nmol/L)				0.066		0.237
No	69	43.5	ref.		ref.	
Yes	327	56.6	1.64 (0.97, 2.79)		1.60 (0.74, 3.46)	
CRP mg/L	419		1.01 (0.98, 1.04)	0.491		
Log transformed CRP mg/L	419		0.98 (0.86, 1.12)	0.793		
CRP >5 mg/L (Women)				0.861		
No	368	54.9	ref.			
Yes	51	52.9	0.95 (0.52, 1.72)			
AGP g/L	419		0.73 (0.36, 1.5)	0.399		
Log transformed AGP g/L	419		0.79 (0.42, 1.47)	0.458		
AGP > 1 g/L (Women)				0.125		
No	364	56.3	ref.			
Yes	55	43.6	0.64 (0.36, 1.13)			
Child age at enrollment (mo)	426		1.08 (1.02, 1.14)	0.010		
Child's sex				0.040		0.348
Boy	257	59.1	ref.		ref.	
Girl	169	49.1	0.66 (0.45, 0.98)		0.8 (0.5, 1.28)	
Breastfeeding status				0.607		
No longer breastfeeding	34	55.9	ref.			
Exclusive breastfeeding	258	52.7	1.11 (0.48, 2.59)			
Predominant breastfeeding	36	52.8	1 (0.38, 2.64)			
Partial breastfeeding	33	45.5	0.82 (0.29, 2.34)			
Continued breastfeeding	65	70.8	1.74 (0.72, 4.21)			
Minimum dietary diversity (Child)						
Minmum meal frequency				0.982		
No	51	68.6	ref.			
Yes	31	67.7	0.99 (0.37, 2.62)			
Minmum acceptable diet						
MUAC (cm)	396		1 (0.87, 1.15)	0.965		
Head circumference (cm)	391		0.96 (0.86, 1.07)	0.423		
Weight (kg)	396		0.97 (0.8, 1.16)	0.711		
Length (cm)	396		0.98 (0.92, 1.03)	0.380		

	N	Anemia prevalence (%)	Bivariate Odds ratio (95% CI)	Bivariate p-value	Multivariable Odds ratio (95% CI)	Multivariable p-value
LAZ	396		0.87 (0.76, 1)	0.046		
WAZ	396		0.9 (0.78, 1.04)	0.147		
WLZ	394		1.06 (0.9, 1.24)	0.516		
Stunted				0.004		0.002
No	266	49.2	ref.		ref.	
Yes	130	66.2	1.9 (1.22, 2.95)		2.25 (1.36, 3.73)	
Wasted				0.711		
No	349	53.9	ref.			
Yes	45	62.2	1.13 (0.58, 2.21)			
Underweight				0.259		
No	284	52.5	ref.			
Yes	112	60.7	1.3 (0.83, 2.04)			
Mean Corpuscular Volume (MCV)	426		0.93 (0.91, 0.95)	0.000		
Age-specific cut-off for low MCV				0.000		0.000
No	326	47.9	ref.		ref.	
Yes	100	79	3.96 (2.23, 7.03)		3.79 (1.89, 7.57)	
Ferritin ug/L	398		1 (1, 1.01)	0.159		
Low ferritin(<12) (Child)				0.144		
No	377	54.6	ref.			
Yes	21	76.2	2.18 (0.77, 6.19)			
sTfR mg/L	398		1.11 (1.04, 1.19)	0.002		
Elevated sTFR(>8.3) (Child)				0.001		0.009
No	307	50.2	ref.		ref.	
Yes	91	74.7	2.68 (1.53, 4.68)		2.48 (1.25, 4.91)	
RBP, corrected (Child)	398		0.76 (0.44, 1.33)	0.338		
Vitamin A deficient- Corrected RBP<0.6 (Child)				0.502		
No	222	54.1	ref.			
Yes	176	58	1.15 (0.77, 1.72)			
EGRAC ratio	343		0.83 (0.64, 1.08)	0.173		
High EGRac (>1.3) (Child)				0.194		
No	99	61.6	ref.			
Yes	244	51.2	0.72 (0.44, 1.18)			

	N	Anemia prevalence (%)	Bivariate Odds ratio (95% CI)	Bivariate p-value	Multivariable Odds ratio (95% CI)	Multivariable p-value
ETKAC	241		1.25 (0.57, 2.74)	0.584		
High ETKac (>1.25) (Child)				0.334		
No	121	50.4	ref.			
Yes	120	55.8	1.29 (0.77, 2.16)			
Blood ThdP nmol/L	280		0.99 (0.99, 1)	0.025		
Low thiamine diphosphate (<95 nmol/L) (Child)				0.066		0.043
No	74	44.6	ref.		ref.	
Yes	206	56.3	1.66 (0.97, 2.87)		2.09 (1.03, 5.57)	
CRP mg/L	398		1.02 (1, 1.03)	0.096	1 (0.97, 1.03)	0.956
CRP >5 mg/L (Child)				0.260		
No	196	52	ref.			
Yes	202	59.4	1.26 (0.84, 1.89)			
AGP g/L	398		1.5 (1.01, 2.22)	0.043	1.61 (0.88, 2.94)	0.120
AGP > 1 g/L (Child)				0.445		
No	237	52.3	ref.			
Yes	161	60.9	1.19 (0.76, 1.85)			

Because of the reduced sample size for thiamine biomarker results, we repeated the multivariable model with and without thiamine outcomes. Multivariable results for thiamine are shown in this reduced sample model, all other results are presented in the full sample size model.
